# Association between sister chromatid exchange and double minute chromosomes in human tumor cells

**DOI:** 10.1186/s13039-015-0192-x

**Published:** 2015-11-19

**Authors:** Jie Xu, Peng Liu, Xiangning Meng, Jing Bai, Songbin Fu, Rongwei Guan, Wenjing Sun

**Affiliations:** Laboratory of Medical Genetics, Harbin Medical University, Harbin, China; Key Laboratory of Medical Genetics, Heilongjiang Higher Education Institutions, Harbin Medical University, Harbin, China

**Keywords:** Double minute chromosomes, Sister chromatid exchange, Double-strand DNA break

## Abstract

**Background:**

Double minute chromosomes (DMs) are the cytogenetic hallmark of extra-chromosomal genomic amplification. They can well represent the advanced stage of malignancy. However, the mechanisms of DM generation are still not fully understood. Here, the sister chromatid exchange (SCE) was used to determine whether the occurrence of DMs was related to the high genomic instability in human carcinoma cells. We analyzed SCE frequencies in two groups of cell lines: the first group contained DM-positive cell lines such as UACC-1598, SK-PN-DW, and NCI-N87 carcinomas, while the second group comprised DM-negative cell lines including HO-8910, U251, and MGC-803.

**Results:**

The data showed that SCE was significantly increased in the DM-positive cells as compared to the DM-negative cells. In addition, there was a positive correlation between the incidence of DMs and the SCE frequency in the UACC-1598, SK-PN-DW, and NCI-N87 carcinoma cells.

**Conclusions:**

Because SCE can reflect general genome instability, it is suggested that the DMs are likely to be closely associated with genomic instability in carcinoma cells. Meanwhile, SCE may be involved in the malignant progression of DM-positive cancers.

## Background

Oncogene amplification is common in tumor cells, and double minute chromosomes (DMs) are the cytogenetic DNA features that harbor those amplified genes, such as C-MYC, MYCN, MDM2, and EIF5A2 [1–3]. Since their first description in the malignant pleural effusion of lung cancer cells, DMs have been found in many human tumors, particularly in solid tumors [4]. DMs are basically small-paired, acentric, and atelomeric extrachromosomal elements [5]. Owing to their close association with gene amplification, DMs have been associated with tumor progression and poor outcomes in malignant cancers. However, the mechanisms of DM generation are still not fully understood. Several hypotheses have been proposed to explain DM generation, and most of the evidence indicates that chromosome breakage is responsible for the development and subsequent amplification of DMs.

Sister chromatid exchange (SCE) is a process in which two sister chromatids break and rejoin during DNA replication and physically exchange regions of the parental strands [6]. Because SCE phenomenon can reflect general genome instability, it is widely used as a reliable and sensitive indicator of chromosome instability [7]. The incidence of SCE was found to be significantly higher in patients with Bloom’s syndrome and Werner syndrome compared with the control groups [8]. In addition, SCE levels are also increased in individuals with cancer diseases, such as ovarian cancer, prostate cancer and breast cancer [9–12]. These diseases are all strongly to be associated with genomic instability.

As a cytogenetic marker of extrachromosomal amplified DNA, DMs are likely to be the result of DNA breakage and incorrect repair [13, 14]. The SCE process is considered to be a mechanism that resolves replication-dependent DNA breakage and repair [6]. Furthermore, following induction, fragile sites that are particularly sensitive to forming gaps or breaks are preferably involved in SCE and implicated in deletions, translocations, and intrachromosomal gene amplification events in cancer cells [15, 16]. Therefore, one can propose that DMs and SCE share common molecular mechanisms that make them closely connected. A recent study has shown association between DMs and increased SCE incidence in infertile patients [17]. However, there is little research on the association between DMs and SCE in tumor cells, and the mechanism involved in the association remains largely unknown.

In this work, by counting SCE numbers, we found that the incidence of SCE was markedly different in tumor cells. Importantly, there was a marked increase in SCE frequency in the DM-positive cells compared to the DM-negative cells. We believe that DMs are strongly connected with SCE in carcinoma cells, and this connection will provide new clues to explain whether, and by which mechanisms, DMs could be related to poor therapeutic effect and poor prognosis of cancers.

## Results

### The frequency of SCE increased in the DM-positive tumor cells

**Fig. 1:**
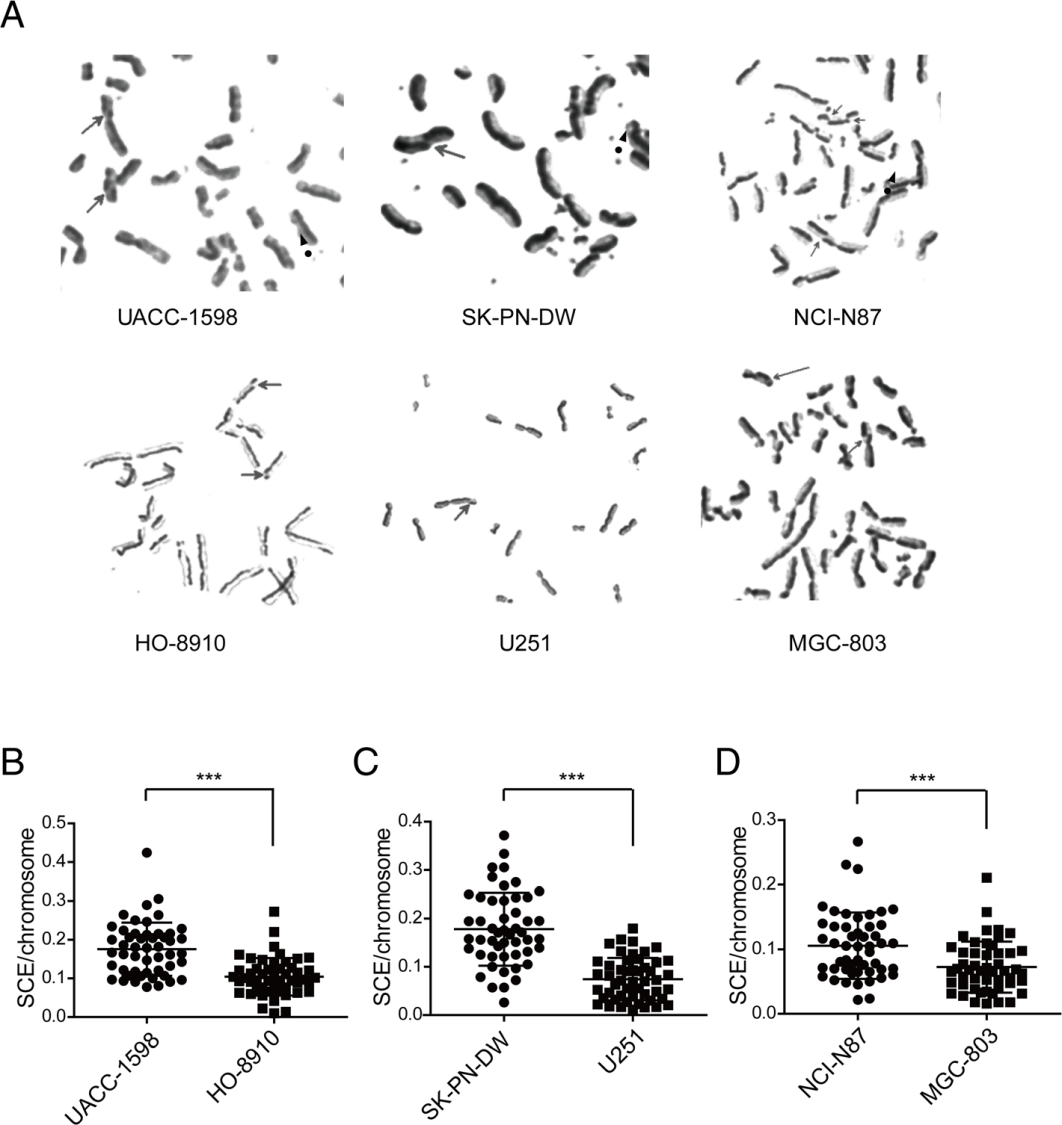
The SCE phenomenon in each cell line. **a**. SCEs were found in both groups of cell lines and are indicated by the straight arrows. The dotted arrows indicate DMs in the DM-positive cells. **b**–**d** The numbers of SCEs are shown for the UACC-1598, HO-8910, SK-PN-DW, U251, NCI-N87 and MGC-803 cells. Data are shown as mean ± standard deviation (SD). *** indicate *P* < 0.001 with *t -test * analysis

Six tumor cell lines, UACC-1598, SK-PN-DW, NCI-N87, HO-8910, U251, and MGC-803 were classified into two groups based on the presence or absence of DMs. Using the FPG method, we found that the SCE phenomenon appeared in all cell lines (Fig. 1a). We observed approximately 50 karyotypes of each cell line and quantified the number of SCE in each cell. We found that in the DM-positive cells, the SCE frequencies were much higher than those in the DM-negative cells (Table 1). With the same tissue origin, the UACC-1598 cells had much higher SCE frequencies than the HO-8910 cells (12.26 ± 5.14 vs. 6.58 ± 3.36), Similarly, SK-PN-DW cells had higher SCE frequencies than the U251 cells (7.40 ± 3.13 vs. 4.16 ± 2.31), and NCI-N87 cells had a higher number of SCEs than the MGC-803 cells (7.00 ± 3.13 vs. 3.84 ± 1.92). In consideration of the chromosome number variant in different tumor cell lines, we counted the number of chromosomes and reevaluated the SCE frequencies relative to the chromosome numbers. We found that in ovarian cancer cells, the relative SCE frequency (×10^−2^) in the UACC-1598 cells with high DMs was significantly higher than that in the HO-8910 cells, which did not contain DMs (17.56 ± 6.79 vs. 10.43 ± 4.81) (Fig. 1b). Similar differential trends were obtained in the neural origin tumor cells SK-PN-DW with DMs and the U251 cells without DMs (17.80 ± 7.47 vs. 7.45 ± 4.35) and in the gastric carcinoma cells NCI-N87 with DMs and the MGC-803 cells without DMs (10.57 ± 5.10 vs. 7.25 ± 3.92) (Fig. 1c, d). The results suggested that high SCE frequency was strongly linked to the incidence of DMs in tumor cells.

**Table 1:** SCE^a^ frequency in DM^b^-positive and -negative groups of tumor cell lines

Source	Cell line	DM incidence	Mean chromosome	SCE frequency
Ovarian tumor	UACC-1598	87 %	71.06 ± 18.01	12.26 ± 5.14
	HO-8910	0	65.28 ± 22.36	6.58 ± 3.36
Nervous tumor	SK-PN-DW	61 %	43.30 ± 13.81	7.40 ± 3.13
	U-251	0	59.66 ± 18.61	4.16 ± 2.31
Gastric tumor	NCI-N87	54 %	68.22 ± 14.97	7.00 ± 3.13
	MGC-803	0	54.88 ± 11.17	3.84 ± 1.92

(The data are the mean number of chromosome or SCE/cell ± standard deviation (SD)

^a^: sister chromatid exchange; ^b^: double minute chromosome)

### Sister chromatid exchange (SCE) is prone to occur on the chromosome long arm

**Fig. 2:**
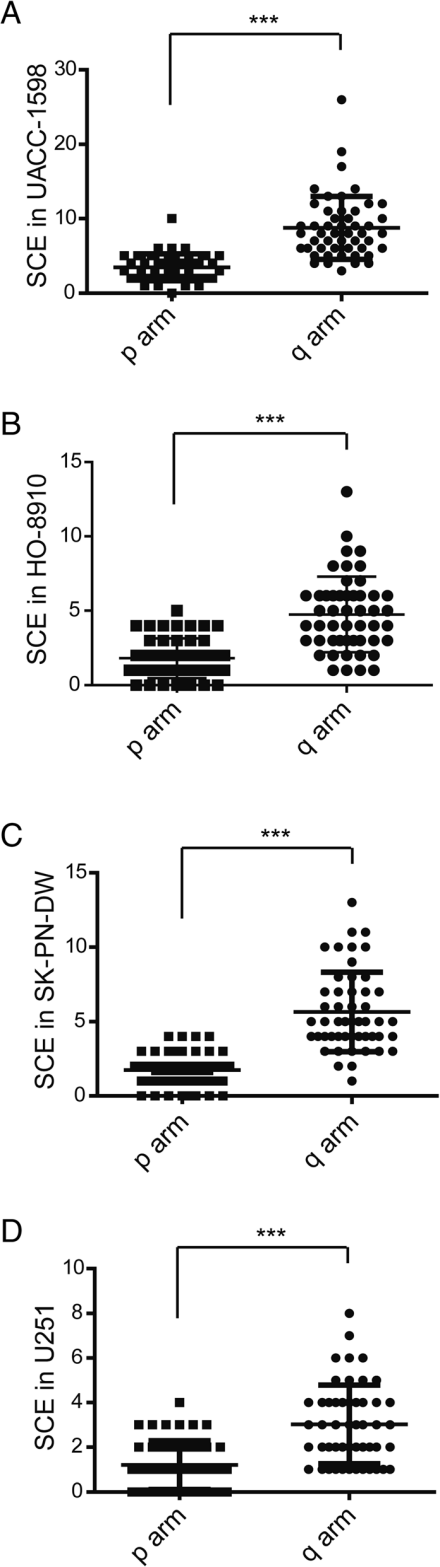
Location of SCE on the chromosome of each cell line. **a**–**d**. The numbers of SCEs on the p arm or the q arm of the chromosome are shown for the UACC-1598, HO-8910, SK-PN-DW, and U251 cells. Data are show as mean ± standard deviation (SD). *** indicate *P* < 0.001 with *t-test * analysis

Furthermore, we recorded and counted the location of SCEs on the p arm and the q arm of chromosomes in the ovarian cancer cells UACC-1598 and HO-8910, as well as in the neural origin tumor cells SK-PN-DW and U251. We found that the SCEs occurred more frequently on the q arm than the p arm in the UACC-1598 (8.76 ± 4.19 vs. 3.48 ± 1.77), HO-8910 (4.76 ± 2.51 vs. 1.82 ± 1.32), SK-PN-DW (5.66 ± 2.65 vs. 1.74 ± 1.14) and U251 cells (3.02 ± 1.75 vs. 1.20 ± 1.08) (Fig. 2a–d).

This indicates that SCEs are prone to occur predominantly on the long arm of the chromosome in both DM-positive and DM-negative cells. In addition, chromosome one and chromosome three were the hot sites for increased SCEs in the UACC-1598 cells; it is in agreement with Simon et al.’s finding that chromosome 1 and chromosome 3 have the highest rates of chromosomal breakage.

## Discussion

In the present study, we found significantly increased SCE frequencies in the DM-positive tumor cells compared with the DM-negative cells. With the same tissue origin, a significantly higher relative SCE frequency adjustment by chromosome number was found in cells with DMs than that in cells without DMs. Our results provide strong evidence that high SCE frequency is closely associated with the incidence of DMs in tumor cells and further our understanding of the function of DMs in tumor malignancies.

The occurrence of genomic instability has been strongly linked to cancer. Various types of chromosomal abnormalities are associated with increased risk of cancer, which includes micronuclei and chromosome aberrations, multicentric chromosomes, ring chromosomes, sticky chromosomes, DMs, as well as sister chromatid exchange [18, 19]. As is widely known, DMs are common phenomenon in a variety of solid tumor cells. They are identified to contribute to cancer evolution by harboring amplified oncogenes and drug-resistant genes [3, 20]. It is well acknowledged that DMs are well reflected an advanced stage of malignancy and regarded as a malignant cytogenetic hallmark [21].

The phenomenon of SCE is the interchange of DNA between replication products that can be visualized in metaphase chromosomes during the S phage of the cell cycle. The SCE process involves DNA breakage and reunification of breaks, which can be induced by various carcinogens, mutagens, or ionizing radiation. Today, the SCE analysis has typically been used as a reliable and sensitive detector of DNA damage, which is simple and rapid for testing chromosome instability [6]. Increased SCE frequency reflects the existence of DNA damage or reduced efficiency in DNA repair mechanism. The increased SCE frequency has been found in individuals with inherited diseases such as Bloom’s syndrome, Werner syndrome and Fanconi’s anemia, all which are known to be associated with genomic instability. More important, several studies have reported an enhancement of SCEs in patients with neoplastic diseases, which includes malignant lymphoma, cutaneous malignant melanoma, lung cancer, uterine cervix cancer, ovarian cancer and breast cancer. It suggests that SCE can also reflect genomic instability and may serve as a preclinical biomarker for early detection of neoplasia.

Currently, DMs and SCE are well known biomarkers for chromosomal instability, but little is known about the relationship between them in human cancer cells. In previous studies, Roy and Zamboni reported that increased SCE frequencies were associated with the appearance of DMs in the mouse methotrexate-resistant clones [22, 23], and the increased SCE levels was reported to be linked with the increase in dihydrofolate reductase (DHFR) activity, which usually amplified on DMs [22]. Therefore, Morgan et al. predicted that SCE frequency would be increased in regions of amplified units (i.e., homogeneously staining regions, HSRs); however, their evidence was failed to support this hypothesis [24]. On the other hand, Winqvist et al. argued that there was no correlation between DMs and SCE because they found an increased frequency of SCE but no extrachromosomal intermediate form of DMs in a colon cancer cell line [25]. In recent study, Papachristou et al. reported that the SCE incidence was markedly greater in DMs-positive infertile patients compared with DMs-negative groups [17]. Our results indicate an intimate association between SCE and DMs in tumor cells by showing that SCE frequencies were significantly increased in DMs-positive tumor cell lines compared with DMs-negative controls and demonstrate a positive correlation between the incidence of DMs and SCE frequency in the DMs-positive tumor cells.

The SCEs identified in our study were predominantly located on the long arm of the chromosomes in both DMs-positive and DMs-negative tumor cells, and chromosomes one and three were the hot sites for SCE incidence in UACC-1598 cells. According to previous report, the distribution of SCEs in a chromosome is not random [26]. Chromosome length is one of the factors that influence the number of SCEs. The longer the chromosome, the more SCEs take place [27]. This hypothesis is corresponded with the results of our study.

## Conclusions

In conclusion, we have shown that DMs are strongly linked to high SCE frequency in carcinoma cells, and this information furthers our understanding of the possible mechanisms of malignancy formation in DM-positive cancer cells. Our findings indicate that the increased incidence of SCE in cancer cells reflects an increased chromosomal instability that may be involved in the malignancy of DM-positive cancers. Thus, SCE is a promising biomarker for assessing the risk of neoplastic progression in DM-positive carcinomas.

## Methods

### Cell lines and cell culture

Three DM-positive tumor cell lines and three DM-negative cell lines were used in this study (Table 2). The UACC-1598 cell line was kindly provided by Dr. Xin-Yuan Guan (University of Hong Kong). The SK-PN-DW and NCI-N87 cell lines were purchased from ATCC (Manassas, VA, USA). The HO-8910, U251, and MGC-803 cell lines were obtained from the Cell Bank of the Chinese Academy of Sciences (Shanghai, China). The UACC-1598, NCI-N87, HO-8910, U251 and MGC-803 cells were cultured in RPMI-1640 medium (Invitrogen, California, USA), and the SK-PN-DW cells were maintained in Dulbecco’s Modified Eagle Medium (DMEM) (Invitrogen), all supplemented with 10 % fetal bovine serum (FBS). All cells were subcultured in the medium containing 5’-bromo-2’-deoxyuridine (BrdU) (Sigma-Aldrich, St. Louis, MO, USA) (at a final concentration of 10 μM) for two cell cycles for differential staining.

**Table 2:** Tumor cell lines

Cell line	Disease	Gender	DM
UACC-1598	Ovarian cancer	Female	Positive
SK-PN-DW	Malignant primitive neuroectodermal tumor	Male	Positive
NCI-N87	Gastric carcinoma	Male	Positive
HO-8910	Ovarian cancer	Female	Negative
U251	Neuroglioma	Male	Negative
MGC-803	Gastric adenocarcinoma	Female	Negative

### Preparation of metaphase spreads

Chromosome preparation was performed using a routine method. Briefly, cells were exposed to colchicine at the final concentration of 0.1 μg/ml for 2 h, and then suspended in 0.075 M KCl at 37 °C for approximately 10 min, followed by fixing with methanol/acetic acid (3:1 (v/v)). After that, the cells were dropped onto cold wet glass microscope slides, and air dried.

### Differential staining for SCE

Genomic SCEs were visualized using standard Fluorescence plus Giemsa (FPG) technique. Slides were allowed to age at 37 °C for 24 h, stained with 0.5 μg/ml Hoechst 33258 (Sigma-Aldrich) in 2 × SSC (0.3 M NaCl, 0.03 M sodium citrate; pH 7.0) at room temperature for 25 min, simultaneously exposed to 365 nm UV light, then incubated in 2 × SSC at 60 °C for 30 min, and finally stained in 2 % Giemsa solution for 25–30 min.

### Data analysis

The SCE frequency was assessed manually from the digital images obtained using a CCD camera coupled to a fluorescence microscope (Carl Zeiss, Baden-Württemberg, Germany). Fifty-second division metaphases were scored by a single observer. The SCE data were analyzed statistically with the Student’s *t*-test using the SPSS program. A probability of *P* < 0.05 was considered statistically significant.

## Abbreviations

SCE: Sister chromatid exchange
DMs: Double minute chromosomes
FPG: Fluorescence plus Giemsa
HSR: Homogeneously staining regions
